# Comparison of hand hygiene in single-room versus open-plan ICUs

**DOI:** 10.1186/cc10684

**Published:** 2012-03-20

**Authors:** I Gork, S Benenson, M Brezis, CL Sprung, PD Levin

**Affiliations:** 1 Hadassah Hebrew University Medical Center, Jerusalem, Israel

## Introduction

In a previous study we showed that cross-contamination with resistant bacteria occurred less frequently in a single-room (SR) ICU when compared to an open-plan (OP) ICU. We attempted to identify whether this was mediated by a change in human behavior; that is, whether hand hygiene (HH) practices were similar in the OP versus SR ICU.

## Methods

The SR ICU comprises eight single-patient rooms. The OP ICU includes four beds in a common area. Covert HH observations were made of physicians and nurses in both ICU areas. Defined HH opportunities occurred before and after contact with the patient or their environment. Each observation session lasted 20 minutes. Compliance was defined as use of alcohol hand rub or chlorhexidine wash. Qualitative records were made of tasks preceding missed HH opportunities (patient contact, computer use, obtaining additional supplies or other).

## Results

Observations sessions were completed on 34 and 35 occasions in the SR and OP ICUs respectively including 277 and 418 HH opportunities. The number of staff observed per session was 2.6 ± 0.7 in the SR ICU versus 2.1 ± 0.5 in the OP ICU (*P *= 0.01). There were fewer HH opportunities per session in the SR ICU (8.4 ± 3.3 vs. OP ICU 11.9 ± 5.2, *P *< 0.001). HH compliance before patient contact was higher in the SR ICU than the OP ICU (1.8 ± 1.4 vs. 0.8 ± 1.1 episodes/session, *P *= 0.001), but similar after patient contact (2.6 ± 1.4 vs. 2.2 ± 1.5 episodes/session, *P *= 0.29). Causes of missed HH opportunities were recorded on 98 and 140 occasions in the SR and OP ICUs. Comparing the SR to OP ICU: patient contact accounted for 21/98 (21%) versus 50/140 (36%, *P *= 0.02) missed HH opportunities respectively; use of the bedside computer 1/98 (1%) versus 14/140 (10%, *P *= 0.005); additional supplies (drugs, cleaning, dressing, and so forth) 9/98 (9%) versus 20/140 (14%, *P *= 0.24); and other 4/98 (6%) versus 15/140 (10%, *P *= 0.06).

## Conclusion

There were more HH opportunities in the OP ICU and HH compliance there was lower. The main difference in compliance occurred before patient care, with compliance after patient care being similar. This may reflect ease of access from patient to patient in the OP ICU where turning around brings you easily from one patient to the next. In the SR ICU movement from patient to patient requires exiting one room and entering another with a clear end to patient care in one room and a beginning in the next. Patient contact and use of the bedside computer accounted for the majority of missed HH opportunities and present possibilities for interventions to improve HH compliance.

